# A peek into the origin of creep in sand

**DOI:** 10.1007/s10035-018-0863-5

**Published:** 2019-01-10

**Authors:** Edward Andò, Jelke Dijkstra, Emmanuel Roubin, Christophe Dano, Elodie Boller

**Affiliations:** 1 CNRS, University of Grenoble Alpes, Grenoble INP, 3SR, 38000 Grenoble, France; 2 Architecture and Civil Engineering, Chalmers University of Technology, SE-41296 Gothenburg, Sweden; 3 ESRF–European Synchrotron Radiation Facility, Grenoble, France

**Keywords:** Sand, Creep, X-ray tomography, Micro-mechanics

## Abstract

This paper presents the results of an experimental study of the particle scale mechanisms that underpin creep, on-going deformations under constant external load, in dry non-cemented sand under 1D oedometric compression loading at 2500 kPa. Traditional observations on the boundary of the sample are complemented with simultaneous measurements of the 3D kinematics of both the entire grain assembly and details of grain-scale mechanisms using synchrotron based X-ray tomography at two different spatial resolutions. Both the continuum response and the local grain scale response are captured using two spatial resolutions, i.e. ${6.5}\,{\upmu }\hbox {m}$ and ${0.65}\,{\upmu }\hbox {m}$ respectively. The results, for the first time, illustrate that small displacements measured at the boundary can be the result of rather pronounced fracturing at the individual grain scale.

## Introduction

The rate-dependent behaviour, such as creep and relaxation, of naturally occurring granular materials is strongly linked to the micro-structure, micro-fractures, crushing and grain surface properties [17, 18, 21, 28]. The general hypothesis in the discipline is that the inter-particle force distribution continues to change under constant external load, probably because of small variations of grain geometrical properties. Bowman and Soga [6] suggest that the distribution of force chains in the assembly play an important role. Two classes of force chains are observed in photoelastic experiments [13] which is corroborated in numerical models [23]. The main force chains, that transmit the majority of the load with only few (often larger) particles, is supported by secondary force chain structures that stabilise the system. Small changes at the grain level will accommodate a redistribution of the load among more grains by initiating some movements in the particle assembly by changes on the grain scale. These local changes could for example be elastic deformation of the grain, plastic deformation at the grain–grain contact, breakage of asperities, or (visco-plastic) sliding at the contact. Lade and Karimpour [19] show, using pre and post mortem particle size analyses, that the time-dependent effects (creep and relaxation) under deviatoric loading in sands originates from particle breakage effects which in turn are linked to static fatigue (failure of material at a stress level below the ‘short-term’ load capacity).

This process is accelerated by the presence of pore water, or more precisely the chemical environment created by the pore water surrounding the grain contacts, that facilitates pressure solution creep. In this process, matter transports through a fluid (film) speeding up weathering and crack growth on the grain–grain contacts. The latter is a mechanism more commonly studied in earth sciences, e.g., [3, 7, 10, 14, 24] where higher stress levels *O(MPa)* and elevated temperatures are studied. Still these effects take place at stress levels far below the failure envelope, i.e., comparable to the static fatigue reported in Lade and Karimpour [19]. Furthermore, continuum observations (traditional displacement measurements at the sample boundary) indicate that the process is grain size dependent, i.e., assemblies of smaller grains lead to significantly larger on-going displacements in the assembly [25].

The process is further complicated by the sensitivity of the observed mechanisms for temperature. Divoux et al. [12] was systematically able to obtain compaction (1% macro strain) in a loose pile of glass beads with 0.5 mm diameter by slowly cycling (with a period of 600 s) the temperature of the system with amplitude variations $\varDelta$T as low as 3 $^{\circ }$C. For studies on pressure solution creep, this temperature sensitivity is addressed by developing a highly regulated test cell using multiple independent systems to control the temperature [15].

Although the micro-structural origin of creep in these materials is undisputed, the exact mechanisms contributing to creep are still largely unknown. Dysthe et al. [14] compares the behaviour of pressure-solution creep on the grain contacts in aggregates of small grains to the classical Andrade creep first developed for metals Andrade [2]. Similar mechanisms are recently postulated for C–S–H bonds in concrete [22]. Unfortunately, the underpinning physical behaviour of Andrade creep is also not fully understood. One plausible explanation, which has some similarities to the theories postulated for granular media, is that under a uniform stress a material with hard and soft regions in the metal or crystal lattice where the local stress deviates slightly from the global stress will generate some strain triggered by a microscopic thermal activation [9].

In this work, multi-scale X-ray tomography observations are used to study creep in an uniform sand in one-dimensional (oedometric) compression at relatively low stress levels ($\sigma _{zz}= {2.5}\hbox { MPa}$). Combined with the temporal resolution offered by the high intensity synchrotron source the effects at the grain and continuum scale can be studied quasi real-time without interrupting the loading.

## Methods and materials

### Test setup

**Fig. 1:**
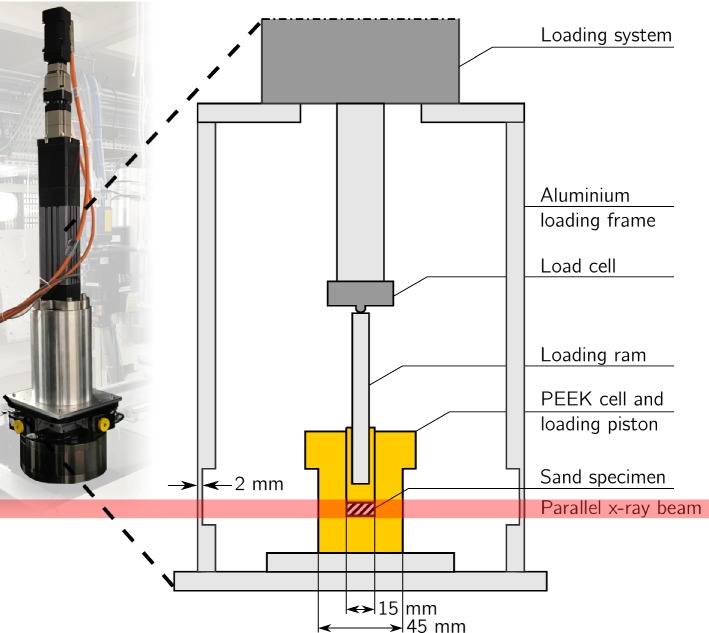
Principal sketch of the loading system used in the experiment with inset of system in place on the id19 beam line rotation stage

We select 1D loading for simplicity of the experimental setup, for the relative ease of reaching stress levels greater than 1 MPa and because the cylindrical specimen shapes are convenient for X-ray tomography. In order to minimise scale effects, (see, for example, Hu et al. [11]), the sample diameter is chosen such that the sample diameter $D_{sample}$ is considerably larger than the mean grain size $D_{50}$. The sample is prepared with a zero drop height, and the top surface is compacted with the loading ram in order to assure a horizontal surface. To minimise wall-friction effects, the sample height $h_{sample}$ is > 5 $D_{max}$. The 1D oedometer cell and loading cap are machined from Polyether Ether Ketone (PEEK) to ensure sufficient stiffness and low X-ray attenuation. A constant axial load is applied using a mechanical actuator capable of applying 15 kN compressive force, with a minimum loading rate of 2 ${\upmu }\hbox {m}/$min. Although no strict load control using a PID loop is employed, the largest ripple in the regulation still is below 0.5% of the desired stress. Force ripples occur most significantly during scanning, where the complete sample stack (which has a rather high centre of gravity) is rotated over $180^{\circ }$ and back. A 500 N load cell (HBM C2-500N) records the applied load. The overall displacement applied by the loading system is taken from the motor’s encoder, relatively far from the specimen itself, meaning that externally measured displacements are an overestimation of the displacement applied to the sample. Figure 1 shows a principal sketch of the test setup.

The microtomography instrument at the id19 beam line at the European Synchrotron Radiation Facility in Grenoble [5, 29] is used for acquisition of the X-ray tomographies in two different modes: a full-sample “low-resolution” (LR) mode with pixel size of 6.5 $\upmu$m and “high-resolution” local tomography within the same sample at 0.65 $\upmu$m. The high flux beam available means that scanning times are relatively rapid (around 5 min for both types of scans). A recent upgrade at ID19 is a dual optics and camera carousel (equipped with 1X and 10X objectives and pco.edge scientific CMOS cameras) for fast switching between the two scanning modes. In brief, the instrument on id19 offers three particular advantages over lab X-ray tomography for this study:

1. Scanning times of a few 100 s.
2. Ability to change modes (i.e., level of zoom) rapidly.
3. Possibility of high-resolution local tomography within a relatively large sample and in-situ setup.

### Test procedure

The tests are performed on a dry, uniform angular sand. The average grain size $D_{50}$ of the sand, classified as Hostun HN31 is 0.328 mm. Dry samples are prepared by pouring the dry material into the test cell whilst tapping the sides. This yields a $D_{sample}$ = 15.00 ± 0.02 mm and $h_{sample}$ = 6.48 ± 0.01 mm, containing 1.7 ± 0.1 g of sand, giving an initial bulk density $\rho$ of 1483 ± 91 kg $\mathrm{m}^{-3}$. Any change in bulk density occurring over the duration of the test falls within this accuracy, given the small vertical displacements, < 3.5 $\upmu$m and the low accuracy of the balance available during preparation of the samples.

The test cell is subsequently mounted on the rotation stage where a first bedding load step of $\approx \,$10 kPa is applied before loading to the target creep load of 2500 kPa at a loading rate of 2.5 $\upmu$m/s until 1500 kPa, 0.25 $\upmu$m/s until 2250 kPa and further reductions in rate until 2500 kPa. The sample is loaded for *circa* 120 min before unloading back to 10 kPa. LR-scans of the full specimen are performed just before creep load application (at 10 kPa), just after the load increment (at 2500 kPa) and again just before unloading (at 2500 kPa) and finally after unloading (at 10 kPa). To track the local grain mechanisms during the creep interval, between the two LR scans at the beginning and end of the creep interval, 10 HR-scans of the local gauge volume are performed with an interval of 10 min over a total period of 100 min.

The LR-scans use a 68 keV beam and collect 2502 projections for each scan. HR-scans required a smaller and high intensity beam of 93 keV, and 1502 projections are collected. In both cases, 3D data is reconstructed using parallel filtered back-projection with Paganin phase retrieval with a $\frac{\delta }{\beta }$ ratio of 500. This reconstruction technique, uses salient phase contrast data to increase contrast, at the cost of a small loss in image sharpness.

### Data analysis

**Fig. 2:**
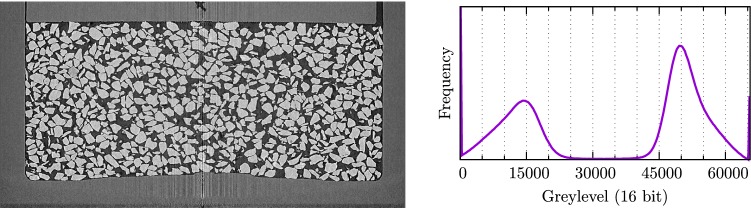
Left: vertical slice LR before creep phase. Right: grey level histogram

After reconstruction, the LR-data is down-sampled with $2\times 2\times 2$ binning yielding a pixel size of 13 µm which reduces noise. Figure 2 (right) shows the well-separated grey level histogram thus obtained.

The LR scan at the beginning of the creep phase at 2500 kPa is segmented using a watershed algorithm [4, 26] that uniquely labels each grain in the volume. Using the newly-developed Spam toolkit, a “discrete correlation” in the style of [1, 16] is performed grain-by-grain using the Digital Volume Correlation (DVC) formulation proposed in Wagne et al. [27], based on Lucas and Kanade [20]. This aims to find a linear transformation operator $\varvec{F}$ which minimises the following greyscale residual. The linear transformation operator describes 3D translations, 3D rotations, and linear strain. The residual essentially the sum of the square differences between the grey levels of the reference configuration before creep (im1) deformed by $\varvec{F}$, and the grey levels of the deformed configuration after creep (im2):

1 $$\varvec{F} = \text {argmin}_{\varvec{G}} \ \sum _{\varvec{x}\in \text {ROI}}\left( \text {im}1(\varvec{G}.\varvec{x}) - \text {im}2(\varvec{x})\right) ^2,$$

where ROI is defined on the mask of a grain. DVC is performed only on the grey levels corresponding to each grain’s label increased by a single dilation. The result of the correlation is obtained by an iterative procedure to solve problem 1, which yields the optimal operator $\varvec{F}$ for each grain. $\varvec{G}.\varvec{x}$ is applied using 5th order interpolation of im1, and the origin of the transformation is defined as the centre of mass of each particle.

In the following, the translation components of $\varvec{F}$ will be shown grain-by-grain, as well as the minimised residual for each grain (calculated afterwards away from the edges of each grain in order to avoid problems at the contacts). Correlations are also performed on the same grains bonded with epoxy in the same scanning conditions, in order to quantify the error on the measured translation components of $\varvec{F}$, yielding a $1\sigma$ error of 0.24 $\upmu$m in displacement, which is considered an upper bound on the error (due to reduced contrast with epoxy compared to air).

The definition of contacts in granular materials is a complex measurement, see Wiebicke et al. [30]. Here we define “potential contacts” in the LR measurements as those where labels are touching in the labelled image—this will therefore overestimate the number of contacts. The position of a potential contact between particles *A* and *B* is defined as the centre of mass of the point cloud defined by the intersection of voxels of *A* in the 6-neighbourhood of *B* and vice versa. Potential contact sliding therefore is computed (in the style of Hall et al. [16]) by applying each measured $\varvec{F_{A,B}}$ to the position of the contact, yielding a displacement vector. The difference in these displacement vectors gives the relative displacement at the potential contact.

Performing measurements of kinematics on the HR-data is not very meaningful due to the relatively large number of incomplete grains in the imaging volume. The tenfold increase in spatial resolution does however yield precious insights into the mechanisms occurring at the scale of individual grains, allowing at this stage occular observation.

## Results

### External measurements

**Fig. 3:**
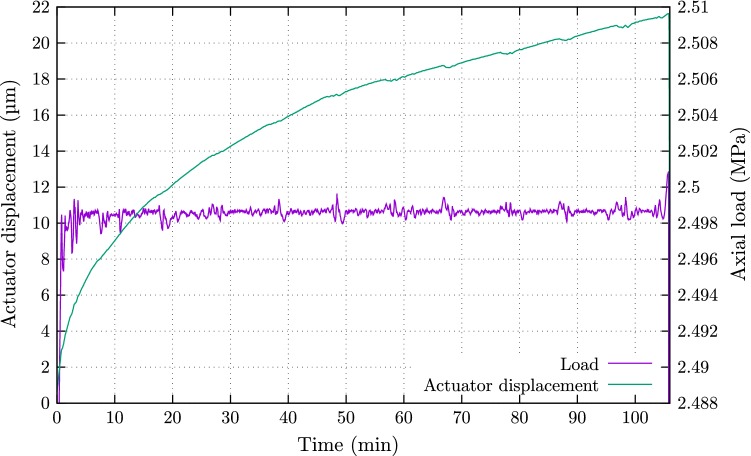
Axial load and displacement evolution against time during creep loading. Slight disturbances (increases in amplitude) at the end of scanning when the rotation stage returns to its initial position rapidly

The load and displacement applied by the actuator are plotted in Fig. 3. Note that the load axis is formatted to show the variations in the load regulation whilst displacements are normalised with respect to the first reading just before the creep stage. The small peaks that occur every 10 min in the load curve relate to the tomography scans. A similar perturbation is found in the displacement data. During the scans the displacements were sufficiently small not to create issues during the local scans. The total vertical displacements, that include deformations in the load piston and cell, are almost 22  $\upmu$m which equates to 0.34% axial strain.

### Grain kinematics

**Fig. 4:**
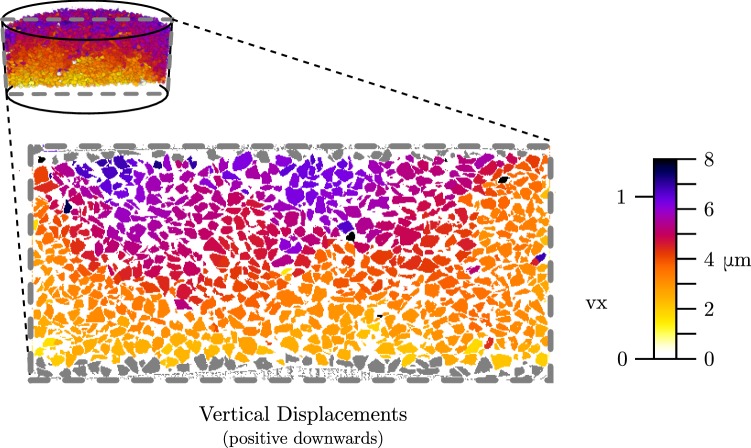
Vertical displacements of grains over the creep phase (with grains touching top and bottom boundaries excluded from tracking and coloured in grey). Loading is from the top of the specimen

The DVC applied to the LR scans acquired under load at the beginning and end of the creep phase (respectively im1 and im2) allow grain displacements to be finely quantified within the specimen. Figure 4 presents a vertical slice containing the axis of the sample where labelled grains are coloured by the vertical component of their displacement, extracted from $\varvec{F}$. Poorly segmented grains touching the top and bottom loading platens are omitted from tracking and are shown in grey.

The grains shown move on average 6 $\upmu$m at the top of the cell and 2.5 $\upmu$m at the bottom, giving an overall axial shortening of around 3.5 $\upmu$m which corresponds to 0.055% axial strain and is much lower than the externally measured 22 $\upmu$m due to the stiffness of the entire loading system. Some traces of wall friction are observed in Fig. 4, with grains in the middle of the specimen moving further downwards under the load, compared to those near the side wall.

**Fig. 5:**
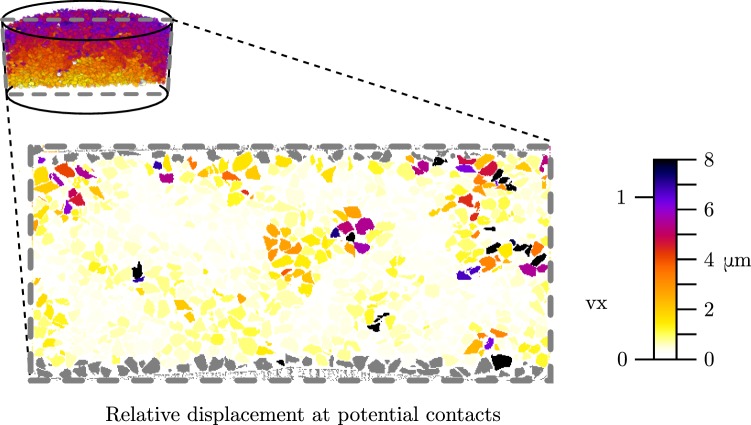
Same slice as above—grains are coloured with respect to their maximum value of relative displacement at the contacts

Progressive sliding at inter-granular contacts is the principal mechanism of irreversible (plastic) deformation in granular materials. Figure 5 illustrates the maximum relative displacement for each grain over each potential contact. It is important to note that the scale bars for displacements are the same between Figs. 4 and 5, indicating that a number of large events occur during the creep test – the source for these large displacements at the contacts is either grain breakage or grain rotation.

### Grain breakage phenomena

**Fig. 6:**
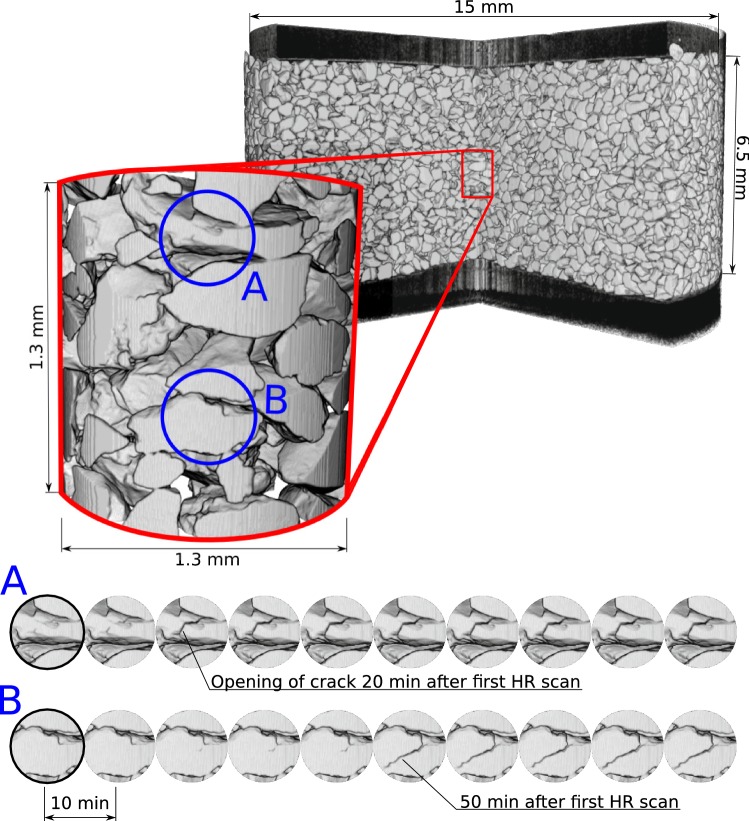
A 3D rendering of a large-field of view scan of the entire sample, with the position of the high-resolution local scans shown as inset. Positions labelled A and B on the initial high-resolution scan are tracked through time with 10 min separating the start of each scan

The ability to perform local scanning at high resolution on the same sample as the LR-scans within minutes opens up the possibility of studying the grain interactions in addition to the global response. Only a relatively small gauge-volume (1.3 mm in height and 1.3 mm in diameter) is captured in the HR-scans. This volume is located at the centre of the sample and is shown in Fig. 6. The Figure also summarises the grain scale analyses, where two local grain breakage mechanisms are highlighted on the initial HR-scan with blue circles *A* & *B*. The time series, with interval 10 min of those two volumes of interest are plotted at the bottom of Fig. 6.

A clear crack opening is already noticed in region *A* after 20 min. Thereafter, another crack in a nearby grain which is directly below in location *B* is observed. This new crack starts approximately 10 min after the crack in *A*. Both grains crack internally through the bulk of the grain. As opposed to crack *B* crack *A* already had a noticeable discontinuity (density) at the start of the time series. Another observation is that crack *A* propagates much faster, basically within 10 min, than crack *B* that takes approximately 20 min to fully progress. The latter, perhaps, indicates that crack *A* is re-activation of an existing crack, whilst crack *B* is fresh. It is postulated that crack *B* is triggered by re-arrangements of force chains in the assembly after cracking of *A*. In absence of direct measurement of force, however, the causality between the two events is not proven, for example this may also be explained by two independent mechanisms that lead to sub-critical crack growth each with a different time scale These two discussed events are not the only crack openings observed in the gauge volume, though best illustrate the mechanisms observed.

The grain breakage clearly visible in the HR gauge volume (in the style of Cil and Alshibli [8]) motivates another look at the LR data set. The significantly reduced spatial resolution means that grain breakage is less easy to identify. In order to help with the detection of fractured particles, the Digital Volume Correlation results are processed further. Despite displacements being measured for the majority of grains shown in Fig. 4, the certainty of grain fracturing observed in the HR data means that the hypothesis of a linear transformation mapping the image of each grain in im1 and im2 is evidently violated. The successfully measured $\varvec{F}$ for each grain are therefore applied to each grain in im1 and the resulting displaced greylevels are then subtracted from im2 (this is simply what is calculated for the residual in the DVC computation before it is squared):

2 $$\delta (\varvec{x}) = \text {im1}(\varvec{F}.\varvec{x}) - \text {im2}(\varvec{x})$$

$\delta$ is then integrated over the volume of the grain as defined by the label (also deformed by $\varvec{F}$), after two cycles of erosion, to avoid observed subpixel overlapping at grain contacts and finally normalised by the volume of integration to give $\varDelta$:

3 $$\varDelta = \frac{1}{\text {size(ROI)}} \sum _{\varvec{x}\in \text {ROI}} \delta (\varvec{x})$$

Opening of fractures within the volume of the grain, i.e. a reduction of the fraction of grains in the gauge volume in im2, will be detected by $\varDelta$ becoming more positive.

**Fig. 7:**
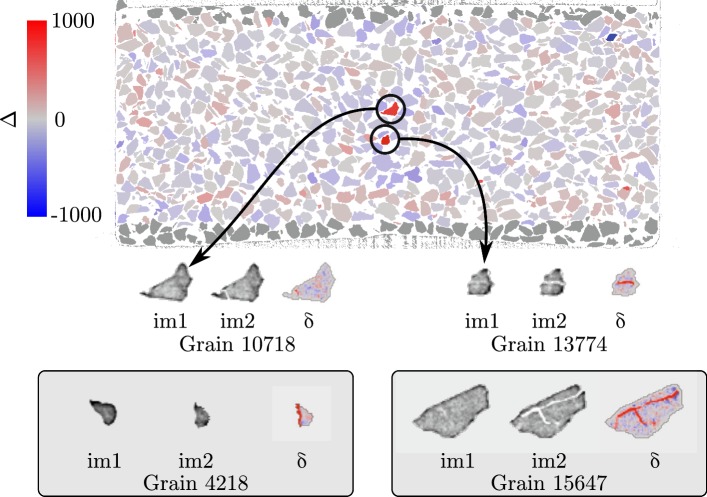
Illustration of DVC-based particle fracture detection. Top: vertical slice though the labelled LR dataset before creep phase (im1) with each grain coloured by $\varDelta$. Sets of three grains illustrate the greyscales corresponding to the mask in im1, in im2 and the $\delta$ field. Grain 15,647 is another grain with high $\varDelta$ which is not present in the labelled slice presented. Grain 4218 is an example of having a high $\varDelta$ which is unfortunately due to to an error in particle tracking

Figure 7 presents a vertical slice through the labelled im1, where grains are coloured by $\varDelta$. The two particles with the highest $\varDelta$ in this slice—which correspond to A and B in Fig. 6—do indeed break. It is important to note that $\delta$ and therefore $\varDelta$ are sensitive to measurement noise both random (Gaussian noise) and structured (unsuccessfully corrected rings for example), for this reason the negative part of the colour bar is also presented in Fig. 7.

The positive side of the colour bar—up to a $\varDelta$ of 1000 should be interpreted with respect to the values of the histogram in Fig. 2, where air has a most-likely value of about 15,000 and grains about 50,000, giving a difference of 35,000 greylevels. Thus a $\varDelta$ of 1000 corresponds to a difference of volume in the reference mask of about 3%.

The field of $\delta$ shown for different grains presents a possible way to detect fractures automatically at this imaging resolution. The number of broken grains—defined as having a $\varDelta$ > 1000—away from the top and bottom platens over the creep phase for the entire specimen is 15 out of the 19,590 grains which are far from the boundaries. This count however is subject to measurement noise as noted above and in the case of $\varDelta$ the volume of integration over each particle, which motivates further study.

## Discussion and conclusions

Dual resolution, i.e., 6.5 $\upmu$m and 0.65 $\upmu$m, high-speed X-ray tomography was applied to monitoring of a 2-h creep experiment in dry sand in oedometric conditions under 2.5 MPa of axial loading. The results clearly illustrate that small displacements measured at the boundary of the sample and in global tomographies of the complete sample volume are in some cases the result of pronounced grain fracturing of a few particles. The monitoring of a local gauge volume shows the progression of large fractures in a few grains, with subsequently breaking grains correlated in space. Finally, a new method for fracture identification based on digital image correlation at the low resolution of 6.5 $\upmu$m compares favourably against the high resolution control measurements. This opens up new avenues for the identification of statistically meaningful data on grain fracture events from low resolution tomographies.
